# Single-Ball Versus Double-Ball Flexor Carpi Radialis Interposition Arthroplasty Following Trapeziectomy: A Retrospective Radiological Study

**DOI:** 10.7759/cureus.117119

**Published:** 2026-09-28

**Authors:** Veeranon Chunyawongsak, Kasidej Jongchanathavorn, Nuttapol Khajonvittayakul, Kittiwan Supichyangur

**Affiliations:** 1 Hand and Reconstructive Microsurgery Unit, Department of Orthopaedic Surgery, Rajavithi Hospital, College of Medicine, Rangsit University, Bangkok, THA; 2 Department of Orthopaedics, Tharongchang Hospital, Surat Thani, THA

**Keywords:** ligament reconstruction, osteoarthritis, tendon interposition, thumb basal joint, trapeziectomy

## Abstract

Introduction

The trapeziometacarpal (TMC) joint represents the second most prevalent site of osteoarthritis in the hand. Following trapeziectomy, ligament reconstruction with tendon interposition (LRTI) is widely considered the gold standard treatment. However, there is currently no objective radiographic evidence comparing single-ball (SB) versus double-ball (DB) flexor carpi radialis (FCR) tendon interposition in preventing first metacarpal collapse after surgery.

Methods

A total of 20 patients who underwent trapeziectomy and LRTI with an FCR graft from 2013 to 2023 were retrospectively analyzed (divided into 10 SB and 10 DB cases), with a minimum radiographic follow-up of one year. Standard posteroanterior radiographs were analyzed to compare preoperative and one-year postoperative parameters, including trapezial void height (HT1 vs HT2), first metacarpal subluxation (MS1 vs MS2), and first metacarpal migration (MM1 vs MM2) measured at baseline and one year postoperatively.

Results

Baseline characteristics did not differ significantly between groups. At one year, the DB group showed a statistically superior trapezial void height (HT2) compared to the SB group (mean difference, −2.98 (95% CI, −5.22 to −0.74); p < 0.05). Conversely, no significant intergroup differences were found in first metacarpal subluxation (MS2) (mean difference, −0.60 (95% CI, −3.45 to 2.25); p = 0.663) or migration (MM2) (mean difference, 2.80 (95% CI, −1.43 to 7.02); p = 0.181).

Conclusions

At one year, the DB technique showed higher absolute trapezial void height than the SB technique. The loss of height from baseline did not differ significantly between groups. Subluxation and migration of the first metacarpal were also similar. Given baseline differences and the lack of functional data, these exploratory results do not establish superior joint space preservation or better clinical outcomes.

## Introduction

One of the most frequent locations for hand osteoarthritis is the trapeziometacarpal (TMC) or thumb carpometacarpal (CMC) joint, which is formed by the articulation between the base of the first metacarpal and the trapezium [1]. Clinical symptoms are most commonly observed in the non-dominant hand of approximately one-third of postmenopausal women over 80 years of age [2], with one-third of this demographic experiencing pain [3].

Despite a relatively high incidence, early-stage disease is rarely symptomatic, leaving most patients untreated initially. As the pathology progresses over time, patients frequently develop substantial impairment of thumb function, grip strength, and overall hand dexterity. The most common contributing factors are ligament laxity, genetics, chronic overuse, and hand injuries [1].

Management strategies for thumb CMC osteoarthritis include both conservative and surgical approaches depending on symptom severity and functional limitation. Conservative management options consist of splinting, nonsteroidal anti-inflammatory drugs, activity modification, and corticosteroid injections [4,5]. When conservative protocols fail, surgical treatment options must be considered, which include joint-preserving procedures when the adjacent scaphotrapeziotrapezoid (STT) joint is normal, or a complete trapeziectomy [3].

For advanced thumb CMC osteoarthritis, trapeziectomy has remained one of the most widely accepted surgical procedures since its introduction in 1949 [3]. Subsequently, various adjunctive techniques were developed to minimize postoperative first metacarpal collapse, which may contribute to residual pain, structural deformity, and thumb weakness. Of these techniques, ligament reconstruction and tendon interposition (LRTI) utilizing the flexor carpi radialis (FCR) tendon continues to be among the most frequently performed surgical interventions [5]. The FCR tendon is commonly selected because its anatomical course resembles the native palmar oblique ligament and it is easily accessible within the standard surgical field. Reconstructive techniques utilizing alternative autografts, including the palmaris longus (PL), extensor carpi radialis longus (ECRL), and abductor pollicis longus (APL), have also been described [4].

Although LRTI continues to serve as an established cornerstone of management for advanced thumb CMC osteoarthritis, considerable variation persists regarding the optimal tendon interposition technique. Tendon interposition configurations vary, ranging from partial FCR autografts, commonly referred to as single-ball (SB) constructs [6,7], to complete FCR autografts configured as double-ball (DB) interposition spacers [8,9]. However, objective radiographic evidence directly comparing their capacities to preserve postoperative trapezial space and prevent metacarpal alignment abnormalities remains limited. To our knowledge, no previous study has directly compared SB versus DB FCR interposition arthroplasty using standardized radiographic parameters. Therefore, the purpose of the present study was to retrospectively compare the short-term radiographic outcomes of these two techniques following trapeziectomy and LRTI, with an emphasis on evaluating the maintenance of trapezial void height, first metacarpal subluxation, and metacarpal migration at one year postoperatively.

## Materials and methods

Study design and patient selection

This retrospective comparative study was approved by the Institutional Review Board (IRB No. 031/2568) of Rajavithi Hospital. Given the retrospective nature of this study, the requirement for informed consent was explicitly waived by the Institutional Review Board. This study evaluated and compared radiographic outcomes between SB FCR interposition arthroplasty with trapeziectomy and DB FCR interposition arthroplasty with trapeziectomy in patients with advanced basal thumb CMC osteoarthritis classified as Eaton-Littler stage III or IV. 

Data were retrospectively collected over a 10-year period from 2013 to 2023. All surgical procedures were performed by a single, highly experienced hand surgeon using a standardized surgical technique to minimize operator variability. The sample size for this study was guided by Vargas-Reverón et al. [8], which included 51 patients diagnosed with basal thumb arthritis. In the present study, a total of 20 patients met all inclusion criteria and were included in the final analysis, divided equally into two groups: 10 patients in the SB group and 10 patients in the DB group. Group allocation was based on chronological transition in surgical practice, where the SB technique was predominantly used during the earlier study period (2013-2017), and the DB technique was adopted during the later period (2018-2023) to enhance interposition volume.

Eligible participants included patients aged 40-70 years with Eaton-Littler stage III or IV thumb CMC osteoarthritis whose symptoms persisted despite more than six months of conservative management (including activity modification, splinting, nonsteroidal anti-inflammatory drugs, and/or corticosteroid injections), and who were deemed fit for surgery under anesthesia. Patients were excluded if they had a history of previous thumb CMC osteoarthritis surgery, ipsilateral hand or forearm fractures, incomplete medical records, or high perioperative surgical risk. Table 1 provides a summary of the baseline clinical and demographic characteristics of both cohorts.

**Table 1:** Baseline demographic and clinical characteristics Data are presented as mean ± SD for continuous variables and as number (percentage) for categorical variables. Continuous variables were compared between groups using the independent-samples t-test. Categorical variables, including sex, operated hand dominance, and Eaton-Littler stage, were compared using Fisher's exact test. A p-value of <0.05 was considered statistically significant. n, number of patients; SD, standard deviation.

Characteristics	Total (n = 20)	Single-ball group (n = 10)	Double-ball group (n = 10)	Test statistic	p-Value
Sex, n (%)				Fisher’s exact test	0.582
Male	4 (20%)	3 (30%)	1 (10%)		
Female	16 (80%)	7 (70%)	9 (90%)		
Age, mean ± SD (years)	65.55 ± 5.97	65.90 ± 7.02	65.20 ± 4.80	t = 0.260	0.798
Operated hand, n (%)				Fisher’s exact test	1.000
Right	9 (45%)	5 (50%)	4 (40%)		
Left	11 (55%)	5 (50%)	6 (60%)		
Eaton-Littler classification				Fisher’s exact test	0.170
Stage III, n (%)	12 (60%)	8 (80%)	4 (40%)		
Stage IV, n (%)	8 (40%)	2 (20%)	6 (60%)		

Surgical technique and postoperative care 

The surgical procedure for LRTI was initiated with a direct skin incision over the radial aspect of the thumb base, followed by a careful capsulotomy of the thumb CMC joint to expose the trapezium bone, concluding with a complete trapeziectomy. Next, according to the surgical cohort (SB or DB technique), the autologous FCR tendon was harvested and prepared. The designated tendon slip was then passed through a pre-drilled bone tunnel at the base of the first metacarpal (M1) bone. Following passage through the bone tunnel, the tendon was tightly sutured back to itself to fill the void created by resection of the trapezium. Finally, a transfixing Kirschner wire (K-wire) was surgically placed to maintain joint stability, support the soft tissue construct, and prevent early postoperative metacarpal collapse (Figure 1). After surgery, the thumb was immobilized in a thumb spica splint with K-wire fixation for six weeks. Following the removal of the K-wire and splint, range-of-motion exercises were started.

**Figure 1:**
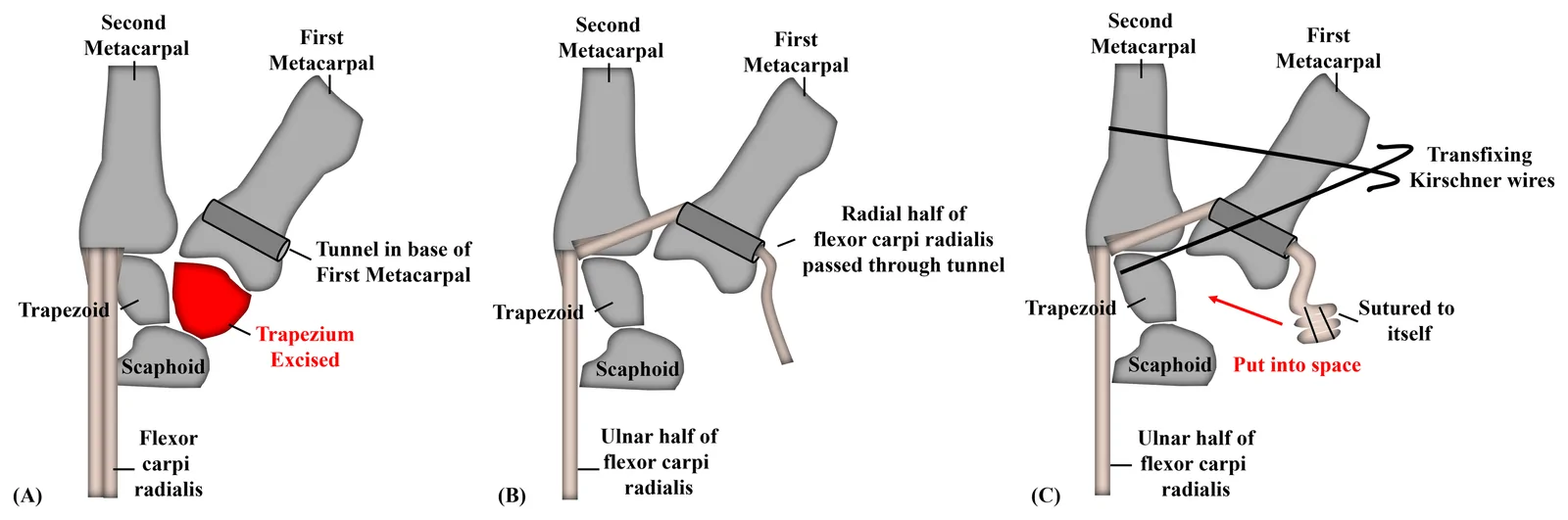
Surgical steps of trapeziectomy and flexor carpi radialis tendon interposition arthroplasty (A) Complete trapeziectomy and creation of a pre-drilled bone tunnel at the base of the first metacarpal bone. (B) Passage of the harvested autologous flexor carpi radialis tendon slip through the first metacarpal bone tunnel. (C) Suturing of the tendon graft to itself within the trapezial void, followed by transfixing Kirschner wire placement for joint stabilization. Illustration created by the authors using Microsoft PowerPoint (Microsoft Corporation, Redmond, WA, USA).

The technical configurations evaluated in this study were operationally defined by the cross-sectional division and structural management of the harvested autograft [6,7,9,10]. The SB group used a longitudinal hemi-tendon split comprising half the cross-sectional width of the harvested FCR tendon to complete the reconstructive construct (Figure 2). In contrast, the DB group used the entire FCR tendon, which was divided longitudinally into two components. Specifically, a strip comprising approximately three-quarters of the tendon's cross-sectional width was passed through the pre-drilled metacarpal bone tunnel and tightly coiled directly inside the trapezial void space. The remaining one-quarter-width strip was placed directly into the empty trapezial space to maximize tissue filling without being passed through the osseous tunnel (Figure 3).

**Figure 2:**
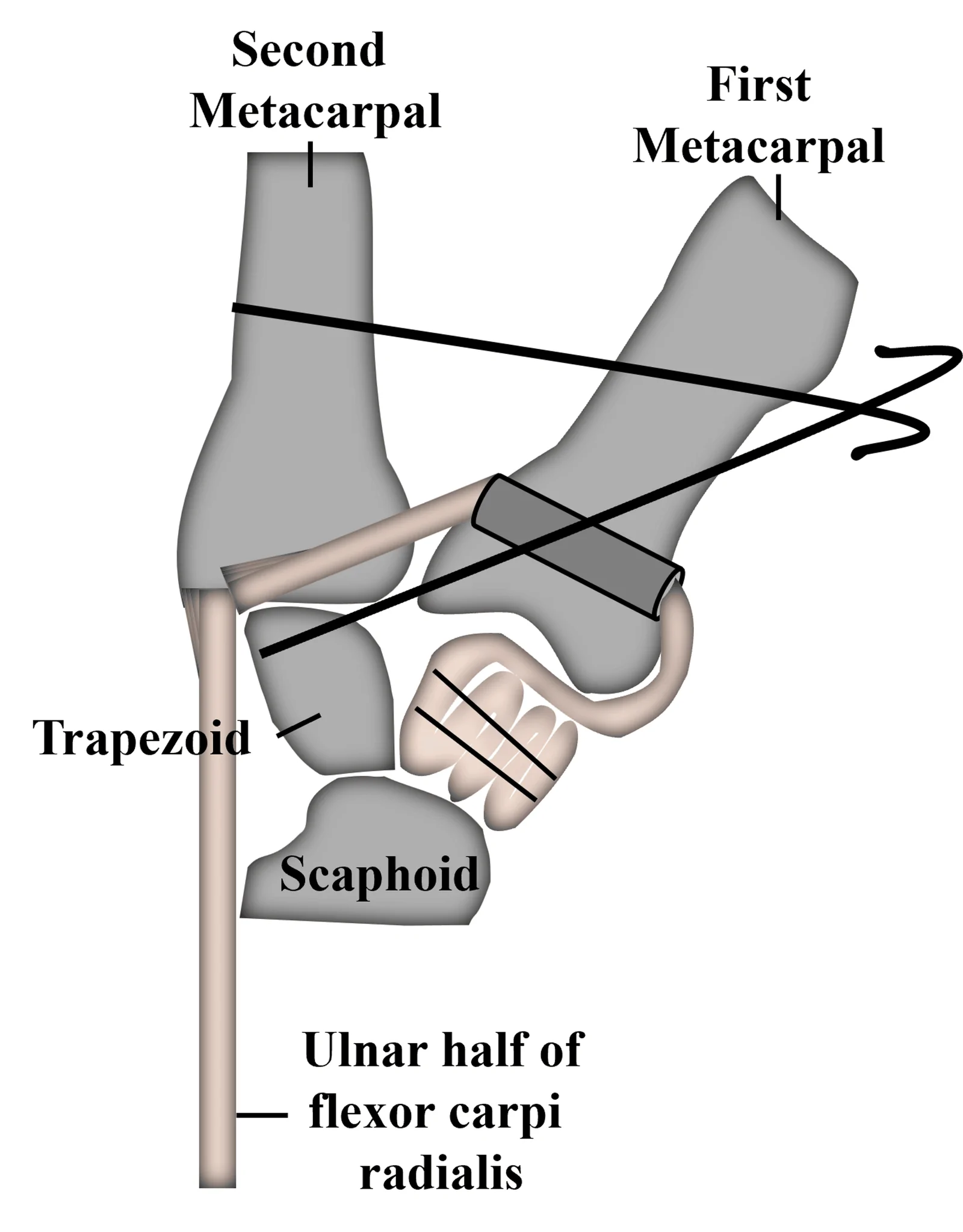
The single-ball flexor carpi radialis interposition arthroplasty technique Illustration created by the authors using Microsoft PowerPoint (Microsoft Corporation, Redmond, WA, USA).

**Figure 3:**
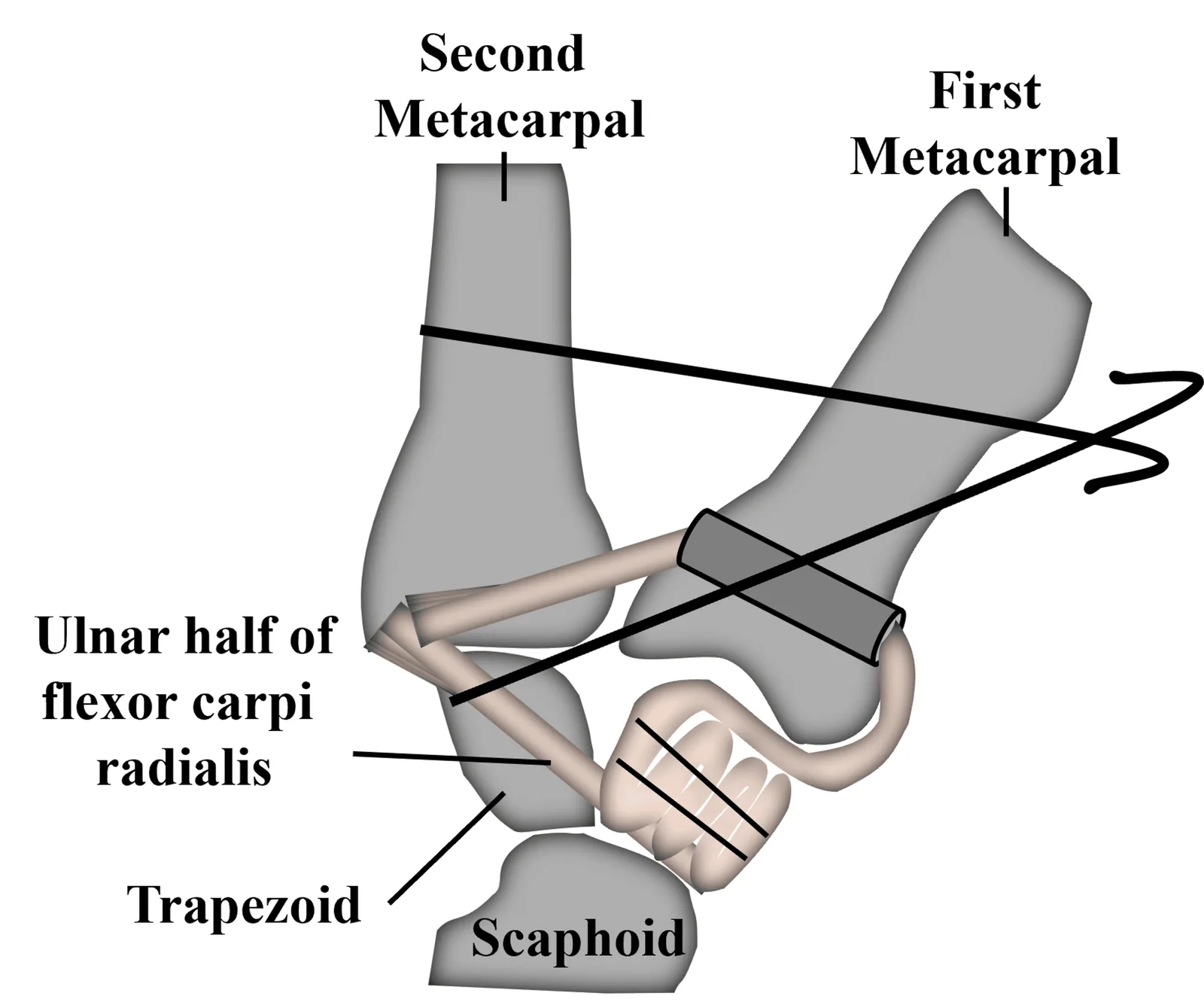
The double-ball flexor carpi radialis interposition arthroplasty technique Illustration created by the authors using Microsoft PowerPoint (Microsoft Corporation, Redmond, WA, USA).

Radiographic parameters

Posteroanterior (PA) radiographs of the involved hand were obtained preoperatively and at the one-year follow-up using a standardized institutional protocol (forearm in neutral rotation, palm placed flat on the detector, and a tube-to-film distance of 100 cm). All digital measurements were performed using the institutional picture archiving and communication system (PACS) calibrated digital measurement tools. All radiographic measurements were performed by an independent orthopedic evaluator who was blinded to patient identity, surgical technique, and clinical records. Measurements were analyzed using the following operational metrics:

Trapezial void height (HT): defined as the direct linear distance measured from the lateral border of the distal articular cortex of the scaphoid bone to the most prominent radial cortex of the articular base of the M1 (Figure 4). Preoperative baseline measurement was designated as HT1, and one-year postoperative measurement was designated as HT2 [11].

M1 subluxation (MS): defined as radial subluxation, quantified as the perpendicular distance from the most prominent radial cortex of the M1 base to a longitudinal reference tangent line contacting both the lateral cortex of the distal radius and the lateral border of the scaphoid (Figure 5). Baseline measurement was designated as MS1, and the one-year postoperative measurement was designated as MS2 [10].

M1 migration (MM): defined as proximal migration, quantified as the perpendicular distance from the center of the M1 articular base to a transverse baseline connecting the proximal cortical bases of the second and fourth metacarpal bones (Figure 6). Baseline measurement was designated as MM1, and the one-year postoperative measurement was designated as MM2 [10].

**Figure 4:**
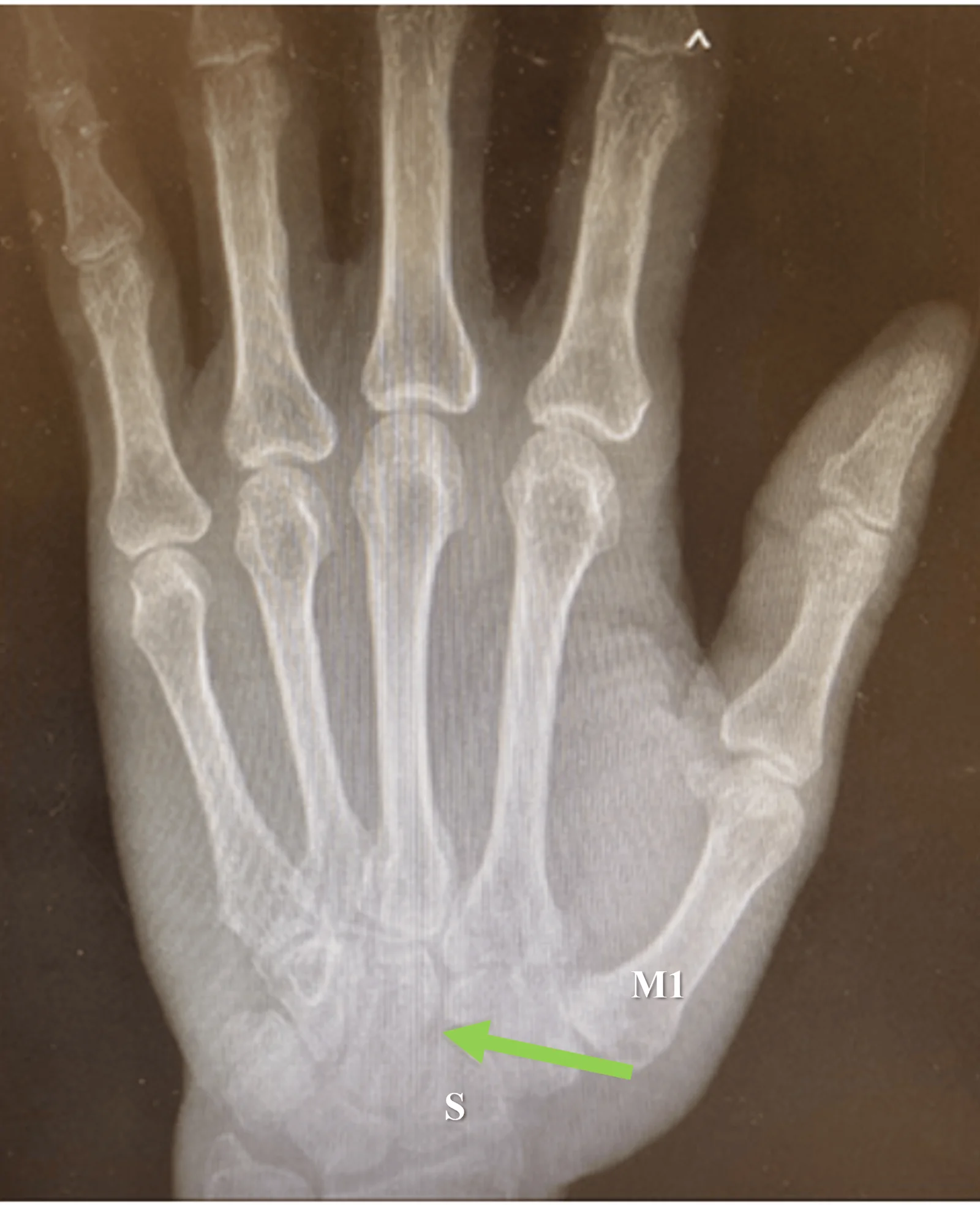
Measurement of trapezial void height on a posteroanterior radiograph The green arrow indicates the linear distance from the distal articular surface of the scaphoid to the base of the first metacarpal. M1, first metacarpal; S, scaphoid. Annotations and graphical markings were created by the authors using Microsoft PowerPoint (Microsoft Corporation, Redmond, WA, USA).

**Figure 5:**
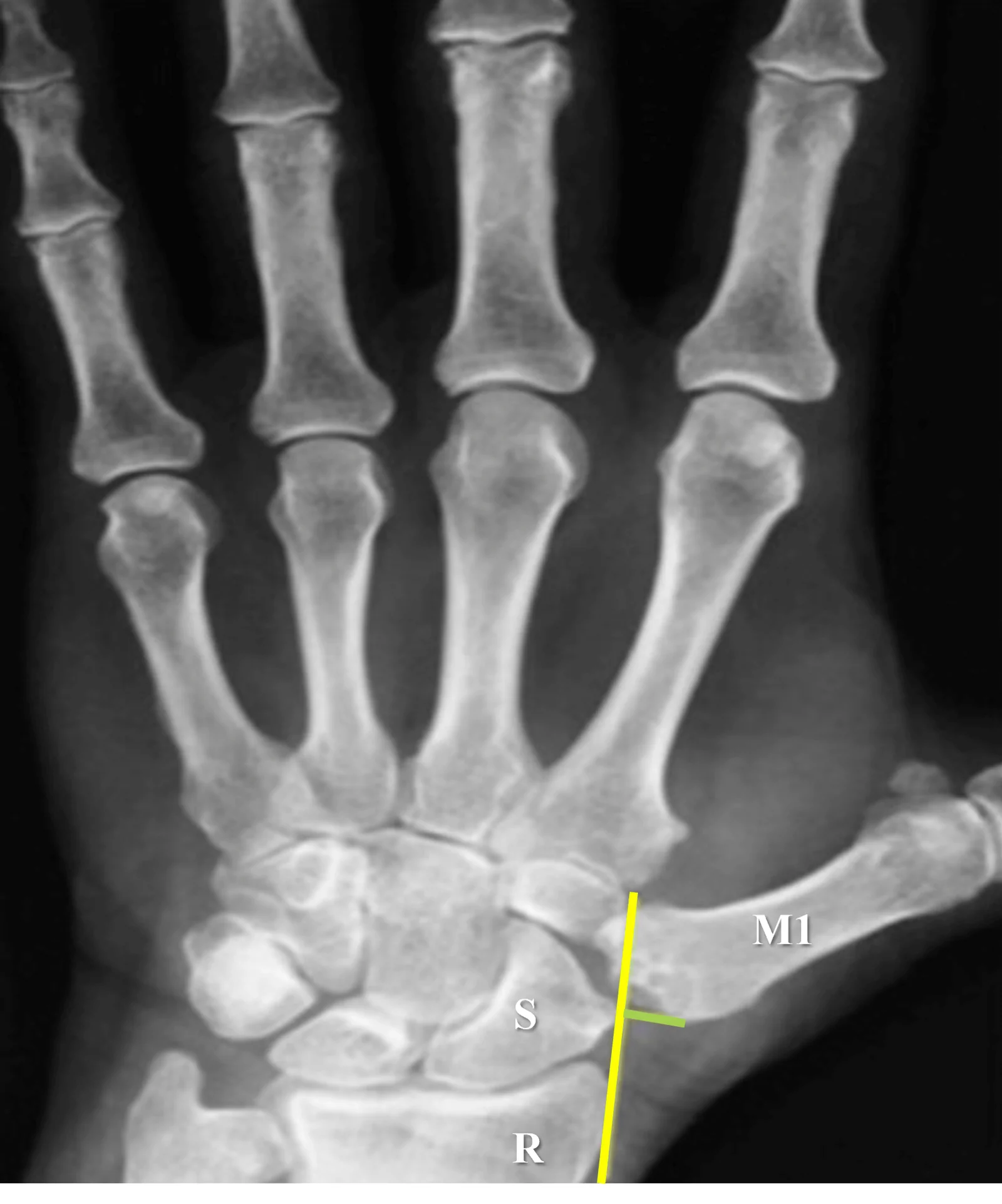
Measurement of first metacarpal subluxation on a posteroanterior radiograph (cropped view) The yellow line represents the reference line drawn perpendicular to a line connecting the outer cortex of the radius to the outer cortex of the scaphoid, and the green line indicates the distance measured from the outer cortex of the first metacarpal to the reference line. M1, first metacarpal; R, radius; S, scaphoid. Annotations and graphical markings were created by the authors using Microsoft PowerPoint (Microsoft Corporation, Redmond, WA, USA).

**Figure 6:**
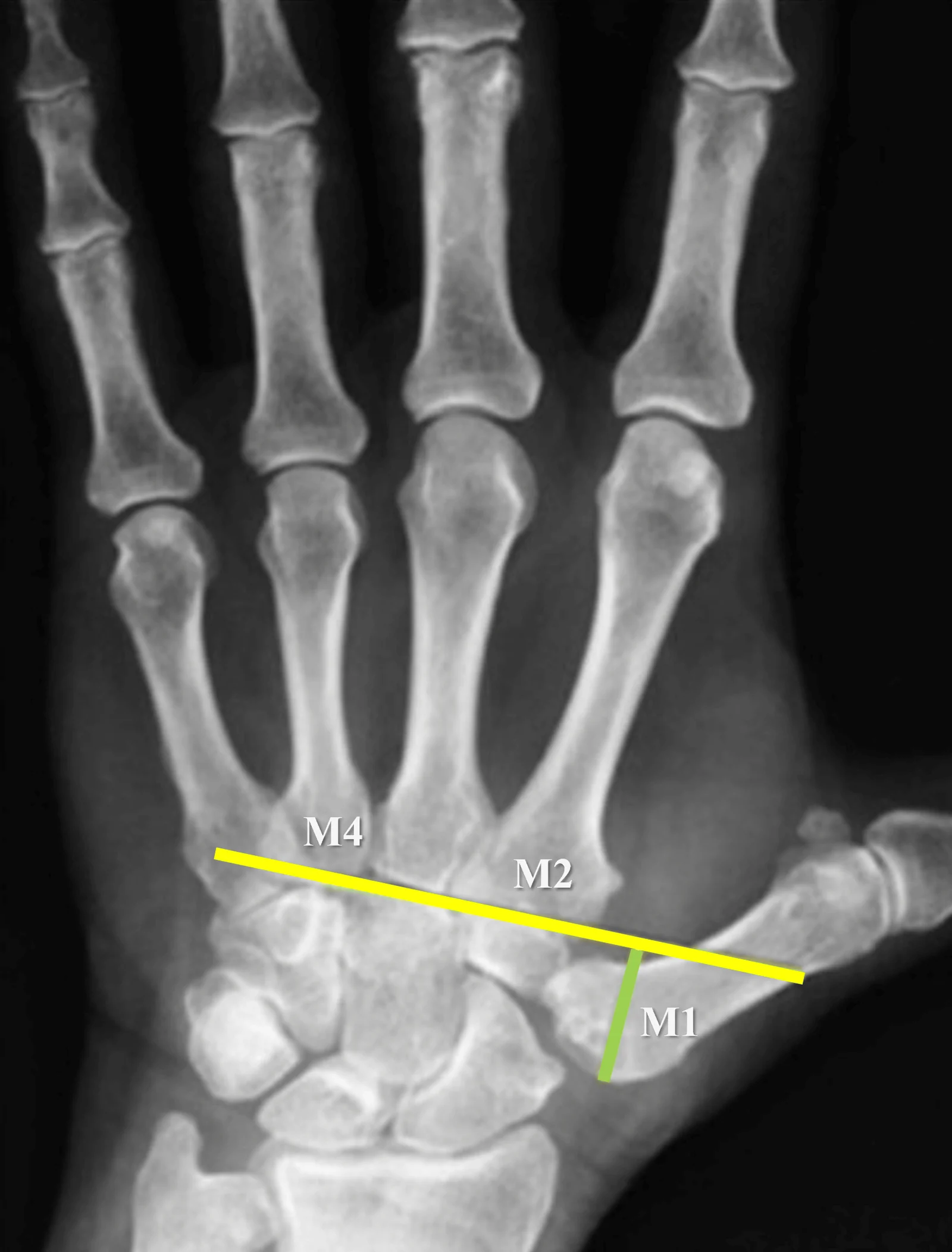
Measurement of first metacarpal migration on a posteroanterior radiograph (cropped view) The yellow line illustrates the reference baseline connecting the second and fourth metacarpal bases, and the green line indicates the perpendicular distance to the first metacarpal base. M1, first metacarpal; M2, second metacarpal; M4, fourth metacarpal. Annotations and graphical markings were created by the authors using Microsoft PowerPoint (Microsoft Corporation, Redmond, WA, USA).

Statistical analysis

Statistical analysis was performed using Stata version 17.0 (StataCorp LLC, College Station, TX, USA) [12]. Descriptive metrics are presented as mean ± standard deviation (SD) for continuous variables and as frequencies and percentages for categorical variables.

For between-group comparisons between the SB and DB groups, continuous demographic and radiographic data (including baseline values, one-year postoperative values, and changes from baseline) were analyzed using the independent-samples t-test. Categorical data distributions were evaluated using Fisher's exact test. Mean differences between the groups with 95% confidence intervals (CIs) were calculated for all primary radiographic outcomes.

The normality of continuous data distribution was assessed using visual examination of quantile-quantile (Q-Q) plots alongside the Shapiro-Wilk test. Because small sample sizes limit the power of formal normality tests, non-parametric Mann-Whitney U tests were also performed as a sensitivity analysis for all primary continuous outcomes to verify the stability of the independent-samples t-test results.

All statistical tests conducted were two-tailed, and a p-value of <0.05 was established as the threshold for statistical significance.

## Results

Data were retrospectively collected over a 10-year period (2013-2023) from patients diagnosed with basal thumb arthritis, Eaton and Littler stage III or IV. Of 22 initially eligible patients, two were excluded due to incomplete medical records. The final analysis comprised a total of 20 patients. These patients were divided into two groups according to the surgical technique received: the SB group (n = 10) and the DB group (n = 10).

There were no statistically significant differences in sex, age at surgery, and operated hand between groups. Eaton-Littler classification distribution showed stage III in eight patients (80%) in the SB group versus four patients (40%) in the DB group, and stage IV in two patients (20%) in the SB group versus six patients (60%) in the DB group (p = 0.170; Table 1).

At the one-year radiographic follow-up, the DB group demonstrated a significantly greater absolute postoperative HT (HT2) compared with the SB group (mean difference: −2.98 mm, 95% CI: −5.22 to −0.74, p = 0.012) (Table 2). The change in HT from baseline (ΔHT) did not differ significantly between groups (mean difference: 1.03 mm, 95% CI: −0.28 to 2.33, p = 0.115). Regarding M1 alignment at the one-year follow-up, the groups showed no statistically significant differences in either MS2 (mean difference: −0.60 mm, 95% CI: −3.45 to 2.25, p = 0.663) or MM2 (mean difference: 2.80 mm, 95% CI: −1.43 to 7.02, p = 0.181) (Tables 3, 4). Similarly, the changes from baseline in MS (ΔMS) and MM (ΔMM) did not differ significantly between the groups (p = 0.260 and p = 0.107, respectively).

**Table 2:** Comparison of trapezial void height between single-ball and double-ball groups at one-year follow-up Data are presented as mean ± SD or mean difference (95% CI). Between-group comparisons were performed using the independent-samples t-test. A p-value of <0.05 was considered statistically significant. CI, confidence interval; HT1, preoperative trapezial void height; HT2, trapezial void height at one year; ΔHT, change in trapezial void height; n, number of patients; SD, standard deviation.

Outcome	Single-ball (n = 10)	Double-ball (n = 10)	Mean difference (95% CI)	Test statistic	p-Value
Preoperative trapezial void height (HT1), mm	13.50 ± 2.82	15.46 ± 2.79	−1.96 (-4.59 to 0.68)	t = -1.559	0.137
Trapezial void height at 1 year (HT2), mm	11.41 ± 2.25	14.40 ± 2.51	−2.98 (-5.22 to -0.74)	t = -2.796	0.012
Change in trapezial void height (ΔHT), mm	2.09 ± 1.58	1.06 ± 1.17	1.03 (-0.28 to 2.33)	t = 1.656	0.115

**Table 3:** Comparison of first metacarpal (M1) subluxation between single-ball and double-ball groups at one-year follow-up Data are presented as mean ± SD or mean difference (95% CI). Between-group comparisons were performed using the independent-samples t-test. A p-value of <0.05 was considered statistically significant. CI, confidence interval; M1, first metacarpal; MS1, preoperative first metacarpal subluxation; MS2, first metacarpal subluxation at one year; ΔMS, change in first metacarpal subluxation; n, number of patients; SD, standard deviation.

Outcome	Single-ball (n = 10)	Double-ball (n = 10)	Mean difference (95% CI)	Test statistic	p-Value
Preoperative M1 subluxation (MS1), mm	7.64 ± 3.25	9.45 ± 3.00	−1.81 (-4.75 to 1.13)	t = -1.295	0.212
M1 subluxation at 1 year (MS2), mm	10.70 ± 0.91	11.30 ± 3.19	−0.60 (-3.45 to 2.25)	t = -0.442	0.663
Change in M1 subluxation (ΔMS), mm	3.06 ± 2.68	1.85 ± 1.91	1.21 (-0.98 to 3.40)	t = 1.163	0.260

**Table 4:** Comparison of first metacarpal (M1) migration between single-ball and double-ball groups at one-year follow-up Data are presented as mean ± SD or mean difference (95% CI). Between-group comparisons were performed using the independent-samples t-test. A p-value of <0.05 was considered statistically significant. CI, confidence interval; M1, first metacarpal; MM1, preoperative first metacarpal migration; MM2, first metacarpal migration at one year; ΔMM, change in first metacarpal migration; n, number of patients; SD, standard deviation.

Outcome	Single-ball (n = 10)	Double-ball (n = 10)	Mean difference (95% CI)	Test statistic	p-Value
Preoperative M1 migration (MM1), mm	8.86 ± 4.00	8.94 ± 2.62	−0.08 (-3.26 to 3.10)	t = -0.053	0.958
M1 migration at 1 year (MM2), mm	14.60 ± 3.21	11.80 ± 5.49	2.80 (-1.43 to 7.02)	t = 1.392	0.181
Change in M1 migration (ΔMM), mm	5.74 ± 2.52	2.86 ± 4.73	2.88 (-0.68 to 6.44)	t = 1.697	0.107

These findings indicate that while patients in the DB group demonstrated greater absolute HT at one year, the degree of height loss from baseline (ΔHT) and M1 alignment parameters were not significantly different between the two cohorts.

## Discussion

This study compared radiographic outcomes between the SB and DB FCR tendon interposition techniques subsequent to trapeziectomy with ligament reconstruction in patients with advanced thumb CMC osteoarthritis. The principal finding was that the DB group demonstrated a significantly greater absolute HT2 compared with the SB group; however, the ΔHT did not differ significantly between cohorts (p = 0.115). To the best of our knowledge, this is the first study to directly compare SB and DB FCR interposition arthroplasty using objective radiographic parameters. Although no significant differences were identified in MS2 or MM2, the DB group maintained greater absolute joint height at one year. Because parameters directly reflecting metacarpal alignment and proximal subsidence (MS and MM) remained comparable between groups, this greater absolute HT cannot be interpreted as definitive evidence of superior structural stability against metacarpal collapse.

Vargas-Reverón et al. [8] evaluated surgical outcomes in 51 patients diagnosed with thumb CMC osteoarthritis. The first group comprised 24 thumbs treated with trapeziectomy and LRTI using an FCR hemitendon according to the Burton-Pellegrini technique [6], whereas the second group consisted of 27 thumbs managed with a modified technique [8]. The modified technique group demonstrated a significantly smaller reduction in the scaphometacarpal space at both one and six months postoperatively (p = 0.0033 and p = 0.001, respectively). The modified approach evaluated by Vargas-Reverón et al. utilized a dual-tendon slip configuration to enhance basal suspension. In our DB construct, the complete FCR tendon was utilized: three-quarters of the tendon width provided ligament reconstruction through the first metacarpal bone tunnel, while the remaining unthreaded one-quarter slip served as an independent spacer within the trapezial cavity. Both studies demonstrate that increasing interposition tissue bulk radiographically reduces immediate postoperative space loss compared with standard hemi-tendon techniques.

Brennan et al. [13] evaluated whether long-term outcomes differed between patients enrolled in a randomized trial comparing trapeziectomy alone with trapeziectomy combined with LRTI. Sixty-five patients were invited for follow-up at a mean of 17 years postoperatively (range: 15-20 years). Twenty-eight patients attended, representing 34 procedures, including 14 trapeziectomy-alone operations and 20 trapeziectomy with LRTI procedures. There were no significant differences between the two groups, including the space between the metacarpal and scaphoid. Radial abduction was significantly increased in the simple trapeziectomy group. LRTI is commonly used to prevent metacarpal subsidence. Long-term studies by Brennan et al. showed that adding tendon interposition does not provide better pain relief or hand function than simple trapeziectomy. The main goal of tendon interposition is to maintain the joint space on radiographs. However, keeping this joint space does not reliably improve clinical outcomes.

Pomares et al. [10] evaluated the clinical and radiological outcomes of trapeziectomy for thumb CMC osteoarthritis in 67 patients treated by the same senior surgeon, with a minimum follow-up of 10 years. The cohort included 16 cases treated with suspensionplasty and 51 with interposition. Clinical outcomes included assessment of strength, pain, and Kapandji opposition score, complications, revision surgery, radiological evaluation, and joint atrophy. The results demonstrated that clinical outcomes remained stable after 10 years of follow-up despite radiological deterioration. Sánchez-Flò et al. [11] conducted a randomized study involving 34 patients with basal thumb osteoarthritis who underwent either partial or total trapeziectomy with interposition arthroplasty. The primary endpoint was key pinch strength at 12 months, and secondary measures included trapezial space height, range of motion, grip strength, change in key pinch strength, and pain. The findings showed that there were no detectable differences between the groups.

Corain et al. [14] conducted a prospective randomized study comparing two surgical techniques following trapeziectomy for stage III or IV TMC osteoarthritis: tendon interposition arthroplasty in 64 patients and K-wire distraction in 56 patients. After a mean follow-up of 6.8 years (range: 3-10 years), greater pain relief was observed in the trapeziectomy with K-wire suspension group, although no significant differences were found between the groups in strength, range of motion, or DASH scores.

From the previous studies, it is evident that the most common surgical approach for patients with thumb basal joint arthritis is primarily LRTI with trapeziectomy. However, the specific tendons chosen for use in each study still vary. The current study was designed to assess short-term radiographic outcomes between the SB and DB FCR interposition techniques following trapeziectomy and LRTI. This research found that the HT2 was significantly better preserved in the DB group compared with the SB group (p = 0.012).

While the DB technique preserved HT better than the SB technique in this study, the clinical significance of maintaining trapezial height remains debated. A well-recognized paradox in trapeziectomy and LRTI literature is the weak correlation between radiographic joint space preservation and clinical outcomes. Therefore, radiographic superiority in trapezial height maintenance cannot be presumed to confer superior pain relief or better hand function.

Several limitations of this study should be acknowledged. First, the retrospective design may have resulted in selection bias, as patient selection for either the SB or DB technique was not randomized and was subject to chronological changes in surgical practice over the 10-year period. A chronological shift in technique could introduce confounding factors, including subtle refinements in surgical skill, alterations in perioperative care, or variations in radiographic acquisition over time. Second, there were baseline differences between the two groups. Stage IV disease was present in 60% of DB patients compared with 20% of SB patients. Baseline HT was also 1.96 mm higher in the DB group (15.46 mm vs. 13.50 mm, p = 0.137). The change in height (ΔHT) showed no significant difference between the groups (p = 0.115). Therefore, the higher postoperative HT2 in the DB group partly reflects this starting difference rather than better joint space preservation. Finally, because the study included only 20 patients, we could not perform analysis of covariance (ANCOVA) or multivariable adjustments without overfitting the model. Third, only radiographic outcomes were evaluated, whereas functional outcomes, pain scores, grip strength, and patient-reported outcomes were not assessed. Because radiographic space preservation does not necessarily equate to clinical success, the true therapeutic advantage of the DB technique cannot be established from these data alone. Fourth, we did not test inter-observer or intra-observer reliability, such as intraclass correlation coefficients (ICC). Our results depend on small millimeter measurements on radiographs. Not testing measurement repeatability is a limitation of this study. Finally, all procedures were conducted by a single surgeon at a single institution, which may reduce the generalizability of the results.

Despite these limitations, this study provides the first direct radiographic comparison between SB and DB FCR interposition techniques using standardized measurement parameters, performed with a uniform surgical technique to minimize operator variability. Future prospective studies with larger cohorts, extended follow-up durations, and comprehensive functional assessments are warranted to determine whether radiographic preservation of HT translates into superior long-term clinical outcomes.

## Conclusions

At one year after surgery, the DB technique was associated with greater absolute HT compared with the SB technique. However, the between-group difference in change from baseline was not statistically significant, and the groups differed numerically in baseline trapezial height and Eaton-Littler stage. No significant differences were observed in MS or MM. These findings are exploratory. These findings are exploratory and do not establish that the DB technique was associated with superior preservation of trapezial height or improved clinical outcomes.
